# Data on Adiponectin from 2010 to 2020: Therapeutic Target and Prognostic Factor for Liver Diseases?

**DOI:** 10.3390/ijms21155242

**Published:** 2020-07-23

**Authors:** Misaq Heydari, María Eugenia Cornide-Petronio, Mónica B. Jiménez-Castro, Carmen Peralta

**Affiliations:** 1 Institut d’Investigacions Biomèdiques August Pi I Sunyer (IDIBAPS), 08036 Barcelona, Spain; misaqheydari@gmail.com (M.H.); cornide@clinic.cat (M.E.C.-P.); 2 Centro de Investigación Biomédica en Red de Enfermedades Hepáticas y Digestivas (CIBERehd), 08036 Barcelona, Spain

**Keywords:** adiponectin, ischemia-reperfusion, liver transplantation, partial hepatectomy, NAFLD, NASH

## Abstract

The review describes the role of adiponectin in liver diseases in the presence and absence of surgery reported in the literature in the last ten years. The most updated therapeutic strategies based on the regulation of adiponectin including pharmacological and surgical interventions and adiponectin knockout rodents, as well as some of the scientific controversies in this field, are described. Whether adiponectin could be a potential therapeutic target for the treatment of liver diseases and patients submitted to hepatic resection or liver transplantation are discussed. Furthermore, preclinical and clinical data on the mechanism of action of adiponectin in different liver diseases (nonalcoholic fatty disease, alcoholic liver disease, nonalcoholic steatohepatitis, liver cirrhosis and hepatocellular carcinoma) in the absence or presence of surgery are evaluated in order to establish potential targets that might be useful for the treatment of liver disease as well as in the practice of liver surgery associated with the hepatic resections of tumors and liver transplantation.

## 1. Introduction

Herein, we review the preclinical and clinical studies reported over the last ten years that investigate the mechanisms of action of adiponectin in liver diseases, in both the presence and absence of surgery. Potential targets and pharmacological strategies aimed at regulating adiponectin in liver diseases are also discussed. Our reasons for this research are threefold. Firstly, there is a need for useful pharmacological strategies to treat nonalcoholic fatty liver disease (NAFLD)/nonalcoholic steatohepatitis (NASH) and avoid progression to cirrhosis and cancer. Secondly, given the prevalence of NAFLD in both society at large and liver transplantation (LT) donation (30% in cadaveric and 20% in living donors), NAFLD pathology has become a major focus of scientific and clinical research [1,2]. Specifically in LT, the presence of NAFLD in the donor liver increases the risk of ischemia/reperfusion (I/R) injury and liver regeneration failure compared with non-steatotic ones. In fact, the use of steatotic livers is associated with an increased risk of primary nonfunction or dysfunction. In addition, many donated livers are not suitable for transplantation because of excess steatosis, which exacerbates the critical shortage of donor livers [3]. Thus, I/R injury associated with LT and hepatic resection of tumors, mostly in different liver diseases, is an unsolved problem in clinical practice. Finally, the increasing number of studies of the role of adiponectin in liver diseases (mainly in the absence of liver surgery) reported in the last 10 years, suggests that this adipocytokine could form the basis of useful strategies for the treatment of NAFLD/NASH and its progression as well as in the clinical practice of liver resections and transplantation.

### 1.1. Characteristics and Isoforms of Adiponectin in Liver Diseases

Adiponectin is a 28 kDa protein hormone including 274 amino acids encoded by the AdipoQ gene. Adiponectin exists in plasma as three distinct oligomeric complexes: the homotrimer (low-molecular weight, LMW mass, ~70 kDa), the hexamer (middle-molecular weight, MMW mass, ~140 kDa) and 12–18 protomer (high-molecular weight, HMW mass, >300 kDa) [4,5].

The total and distribution of all three adiponectin isoforms (LMW, HMW and MMW) are decreased in NAFLD patients without any surgical intervention [6]. HMW adiponectin suppresses growth factor-induced hepatic stellate cell (HSC) proliferation and may be closely associated with lipid metabolism [7]. Experimental data suggest that HMW adiponectin is the most potent isoform for alleviation of fatty liver disease in high fat diet-induced obese mice [8], whereas Bianchi et al. concluded that there is no significant contribution of adiponectin isoform distribution to the progression of liver diseases [6]. In surgical conditions such as normothermic hepatic ischemia associated with hepatic resection, HMW is the predominant isoform of adiponectin in steatotic livers [9]. No data have been reported on the levels of the different adiponectin isoforms in other I/R conditions, such as hepatic resection under vascular occlusion or LT.

### 1.2. Source of Adiponectin in Liver Diseases

Different studies of liver diseases related to NAFLD/NASH without any surgical intervention have suggested that adipose tissue is a major site of endogenous adiponectin production [10,11,12,13,14,15]. However, this situation might be different when patients with NAFLD/NASH require liver surgery. In fact, data obtained from steatotic livers subjected to warm hepatic ischemia without resection as well as from non-steatotic livers undergoing 6 h of cold ischemia without brain death indicate that adiponectin is generated by the liver [9,16]. Indeed, steatotic livers have been shown to generate adiponectin in an isolated perfused liver model without the presence of either circulation or adipose tissue. This is in line with clinical studies reporting that the expression of hepatic adiponectin in patients with NAFLD undergoing partial hepatectomy is derived from hepatocytes themselves rather than from migration of peripheral adipose tissue [17]. Further studies are necessary to elucidate the role of adipose tissue and liver in the changes in adiponectin levels in LT with extended criteria donors (for instance, liver grafts from cardiac arrest donors) as well as in living-related LT.

### 1.3. Adiponectin Receptors in Liver Diseases

Adiponectin enhances the binding of APPL1 (an adaptor protein containing a pleckstrin homology domain, a phosphotyrosine binding domain and a leucine zipper motif) to the two adiponectin receptors (adipoR1 and adipoR2). These interactions are essential for subsequent adiponectin actions [18,19]. However, there is controversy concerning the expression of the adiponectin receptors in animal models of obesity; similarly, hepatic adiponectin receptor mRNAs in NAFLD/NASH patients are found unchanged, decreased or even increased [20,21,22,23,24]. Intensive research efforts are needed to evaluate the expression and role of the different adiponectin receptors in hepatic resections and LT. Nevertheless, given the NAFLD/NASH results, in our view, the role of adiponectin depends on the different isoforms and receptors involved as well as on the type of liver and surgical conditions (as discussed in Section 5 and Section 6 of the current review). If that were the case, specific strategies should be adopted to regulate the effects of adiponectin depending on the type of liver as well as the surgical conditions.

## 2. Adiponectin Effects on Hepatic Damage and Regenerative Failure Associated with Hepatic I/R

**Figure 1:**
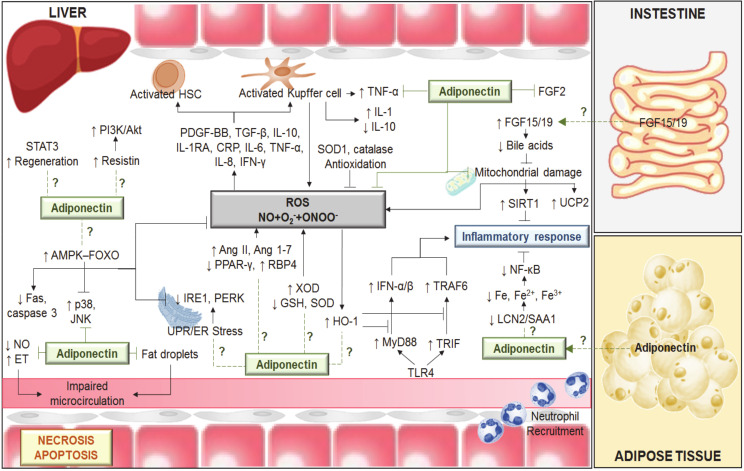
Mechanisms involved in the effects of adiponectin in different liver diseases including hepatic I/R. Ischemia-induced energy deficiency results in the failure of active transmembrane transport and consequently in endothelial cell (EC) and Kupffer cell (KC) swelling. KC activation results in reactive oxygen species (ROS), tumor necrosis factor alpha (TNF-α) and interleukin (IL)-1 release. In addition, ROS (derived from xanthine/xanthine oxidase (X/XOD) and mitochondria), the low levels of antioxidants (superoxide dismutase (SOD), glutathione (GSH) and catalase) and the alterations in the levels of angiotensin (Ang) II, Ang 1–7, peroxisome proliferator-activated receptor-gamma (PPAR-γ), retinol binding protein 4 (RBP4), tumor growth factor beta (TGF-β), IL-1, among others, induce activation of hepatic stellate cells (HSC) and KCs, which in turn induces the release of TNF-α and IL-1 and promotes low IL-10 levels. The imbalance between nitric oxide (NO) and endothelin (ET) production contributes to the narrowing of sinusoidal lumen. The endoplasmic reticulum (ER) stress involves the activation of ER proteins, namely inositol-requiring enzyme 1 (IRE1), protein kinase-like endoplasmic reticulum kinase (PERK) and activating transcription factor 6 (ATF6). ER stress induction contributes to inflammatory response, which might be regulated by adiponectin. This adipocytokine activates the adenosine monophosphate-activated protein kinase (AMPK) ≠ forkhead box protein O (FOXO) signaling axis, showing anti-apoptosis actions, because of decreasing the expression of Fas-associated death domain (Fas) and caspase 3. Adiponectin prevents the activation of c-Jun N-terminal kinase (JNK) and p38 mitogen-activated protein kinase (p38). The anti-inflammatory actions of adiponectin are mediated by a regulation of toll-like receptor 4 (TLR4) signaling via myeloid differentiation primary response gene 88 (MyD88) and TIR-domain-containing adapter-inducing interferon-β (TRIF) pathways; the mitochondrial dysfunctions and the lipocalin-2/serum amyloid A1 (LCN2/SAA1)-iron metabolism. Indeed, adiponectin upregulated the uncoupling protein 2 (UCP2), catalase, and SOD1. Adiponectin might play a crucial role regulating the farsenoid-X receptor (FXR)-fibroblast growth factor 15 (FGF15) pathway in the gut–liver axis. Adiponectin might activate signal transducer and activator of transcription-3 (STAT3) and resistin signaling as well as negatively regulate the fibroblast growth factor 2 (FGF2) response. Akt, protein kinase B; CRP, C-reactive protein; Fe, Iron; HO-1, heme oxygenase-1; IFN, interferon; NF-κB, nuclear factor kappa-light-chain-enhancer of activated B cells; O_2_, superoxide; ONOO-, peroxynitrite; PDGF-BB, platelet-derived growth factor-BB; PI3K, phosphoinositide 3-kinase; SIRT1, sirtuin 1; TRAF6, TNF receptor-associated factor 6; UPR: unfolded protein response. ↑: increase; ↓: decrease.

As mentioned above, the data suggest that the role of adiponectin depends on the surgical conditions and type of liver. Thus, adiponectin is injurious to steatotic livers submitted to 60 min of normothermic ischemia [9]. However, beneficial effects of adiponectin on regeneration are reported in steatotic livers undergoing partial hepatectomy under vascular occlusion. Moreover, adiponectin plays a minor role in non-steatotic liver grafts subjected to 6 h of cold ischemia, whereas it protects in the presence of steatosis [16]. Similarly, to these surgical findings, different results have been reported for the mechanisms of action of adiponectin. It is a positive regulator of resistin in LT of steatotic grafts. Thus, adenosine monophosphate-activated protein kinase (AMPK) activation by the drug, aminoimidazole-4-carboxamide ribonucleoside (AICAR) and surgical ischemic preconditioning increased adiponectin, which in turn upregulated resistin in steatotic liver grafts without brain death. The upregulation of phosphoinositide 3-kinase (PI3K)/protein kinase B (Akt) induced by resistin protected steatotic livers against damage [16]. Meanwhile, data on chronic feeding of high-fat diets to rodents indicate a negative correlation between adiponectin and resistin (decreased adiponectin and increased resistin plasma levels) [25], whereas, in NAFLD patients, no correlation between adiponectin and resistin was reported [26,27]. Furthermore, in preclinical studies without brain death, ischemic preconditioning increased adiponectin levels in liver grafts submitted to 6 h of cold ischemia, but not in LT from brain-dead donors, which most closely resemble the surgical conditions occurring in clinical practice [28]. Furthermore, adiponectin is beneficial for liver regeneration in hepatic resection under vascular occlusion. In fact, results indicate that adiponectin –/– mice exhibit delayed liver regeneration following partial hepatectomy [29,30]. These benefits of adiponectin might be mediated by signal transducer and activator of transcription-3 (STAT3) signaling and progression through the cell cycle. In addition, adiponectin negatively regulates the fibroblast growth factor 2 (FGF2) response and other growth factors derived from stellate cells [31,32] (Figure 1).

## 3. Anti-Steatotic Effects of Adiponectin in Liver Diseases

In obesity, it has been reported that adiponectin protects liver from steatosis and inflammation: it increases the capacity of insulin to suppress glucose production [33]. A diet deficient in choline and L-amino acid induces more severe hepatic steatosis in adiponectin deficient mice than in wild-type animals [34]. In the same way, adenoviral expression of adiponectin ameliorates lipid deposition in the liver. Sterol regulatory element-binding protein 1c (SREBP-1c) is a central regulator of fatty acid synthesis, and it is suppressed by adiponectin in hepatocytes and in the liver of db/db mice [35]. In addition, through the downregulation of lipogenic transcription factor, SREBP-1c, adiponectin prevents hepatic lipogenesis [36]. The induction of AMPK by adiponectin might explain its effects on anabolic and catabolic pathways. It is known that AMPK switches on adenosine triphosphate (ATP)-producing catabolic pathways (fatty acid oxidation and glycolysis) and switches off ATP-consuming anabolic pathways (lipogenesis) [37]. Thus, when adiponectin activates AMPK, glucose utilization and fatty-acid oxidation in the liver are elevated. Suppression of SREBP-1c by adiponectin is mediated through AdipoR1/liver kinase B1 (LKB1)—an upstream kinase of the AMPK pathway [35]. In addition, AMPK phosphorylates acetyl-CoA carboxylase (ACC), and this is subsequently associated with higher activity of carnitine palmitoyl- transferase 1 (CPT-1), a rate limiting enzyme in fatty acid oxidation [37]. In addition, it is reported that adiponectin stimulates peroxisome proliferator-activated receptor alpha (PPARα) activity, which enhances fat oxidation, reduces lipid synthesis and prevents accumulation of fatty infiltration [38]. All of this has been reported in experimental obesity models without any liver surgery.

It should be also noted that adiponectin reduces chronic inflammation in target organs including vasculature, lung and heart, thereby leading to protection against various obesity-related disorders [39]. However, the experiments were performed in vitro so further studies are required to clarify this issue. It has also been demonstrated that serum has lower adiponectin levels, before and after transplantation. However, if such changes are associated with cardiovascular events in long-term follow-up of liver transplant recipients remain to be elucidated [40]. It is also important to clarify this because systemic inflammation following the release into circulation of different mediators from the liver has been reported in surgical conditions of hepatic I/R [41,42].

**Figure 2:**
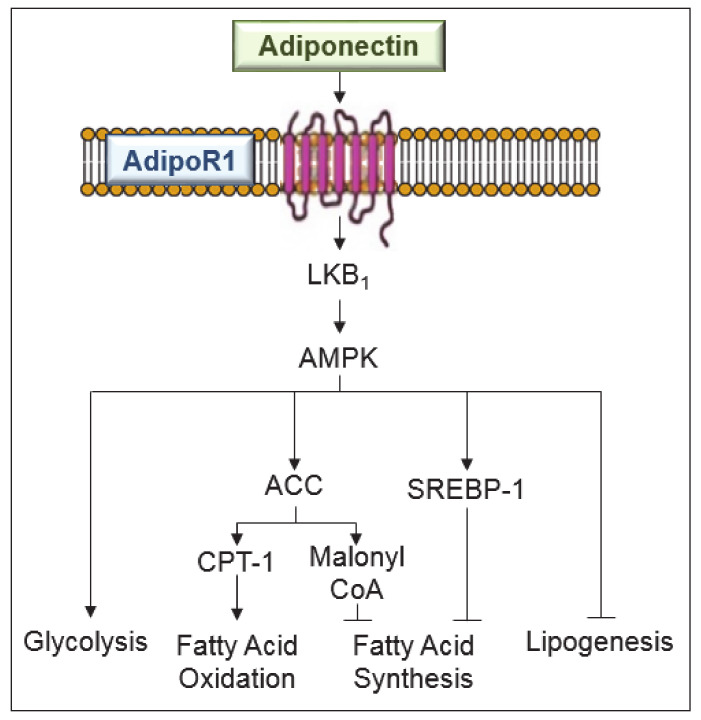
Signaling pathways involved in the anti-steatotic effects of adiponectin in liver diseases. Adiponectin, mediated by adenosine monophosphate-activated protein kinase (AMPK) exerts the following effects: suppresses sterol regulatory element-binding protein-1c (SREBP-1c), a central regulator of fatty acid synthesis and an inhibitor of lipogenesis; increases the glucose utilization and fatty-acid oxidation; increases the activity of carnitine palmitoyl-transferase-1 (CPT-1), a rate limiting enzyme in fatty acid oxidation; and regulates malonyl CoA for fatty acid synthesis inhibition. ACC, acetyl-CoA carboxylase; LKB, liver kinase B1.

The effects of adiponectin in steatotic livers undergoing surgery and on various obesity-related disorders should not be ignored. Indeed, the different signaling pathways such as AMPK and PPARα involved in the anti-steatotic effects of adiponectin also play a role in protecting steatotic livers undergoing I/R against damage (Figure 2).

## 4. Relationship between Adiponectin and Leptin in Fibrogenesis

Hepatic fibrosis is the last prevalent pathway prior to cirrhosis and eventually requires LT. Among the different adipocytokines (retinol binding protein 4 (RBP4), resistin, apelin, chemerin, vaspin, etc.), adiponectin is one chiefly involved in hepatic fibrosis [43]. In patients with liver cirrhosis, circulating adiponectin is elevated, independent of disease etiology, age or body mass index [44]. This might be explained by its effect on the inflammatory response [45], a reduction in biliary excretion [46] or an imbalance between adiponectin production and hepatic excretion [47]. Both leptin and adiponectin play key roles in obesity-related disorders and are associated with the pathogenesis of NAFLD [48]. In NAFLD patients, concentrations of adiponectin are decreased whereas leptin levels are increased, indicating an imbalance of adipocytokines, which might promote the progression of this liver disease [48]**.** Indeed, in vitro studies based mainly on culture-activated HSCs suggest that hepatic fibrosis and its resolution are controlled by adiponectin and leptin.

Adiponectin has strong-antifibrotic features whereas leptin acts as a profibrogenic molecule. This is because adiponectin prevents leptin signal transduction [43]. In fact, adiponectin (through protein tyrosine phosphatase 1B (PTP1B)) prevents the leptin-mediated activation of the janus kinase 2 (Jak2)/STAT3 pathway. By inducing suppressors of cytokine signaling 3 (SOCS3), adiponectin also suppresses leptin activity. Moreover, adiponectin provokes HSC apoptosis and suppresses HSC proliferation and α collagen biosynthesis. In addition, adiponectin-mediated prevention of leptin signaling leads to downregulation of tissue inhibitor of metalloproteinase 1 (TIMP-1) transcription and TIMP-1 activity, while adiponectin increases the capacity of matrix metalloproteinase 1 (MMP-1) to degrade fibrillar collagen in cellular matrix. It further prevents the activity of focal adhesion kinase (FAK) and disrupts the formation of mature focal adhesions (FA) [43]. All of this is scientifically and clinically relevant, but several concerns should be taken into account: it all needs to be confirmed in an experimental model of NASH that simulates the characteristics of this pathology as soon as possible in clinical practice.

**Figure 3:**
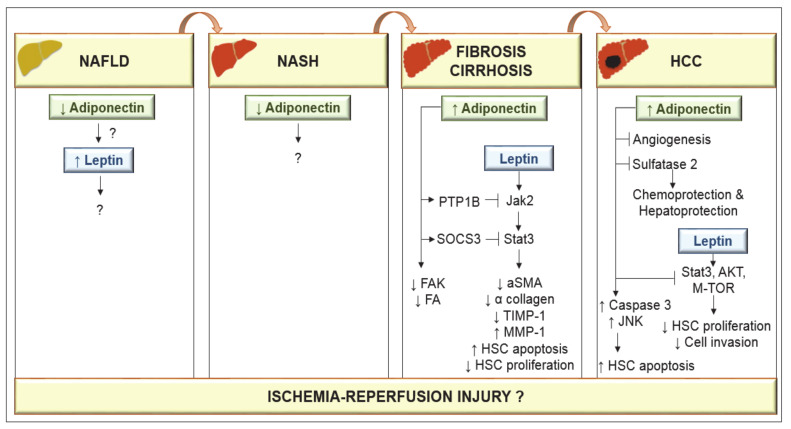
Adiponectin and leptin in the progression of nonalcoholic fatty liver disease (NAFLD) to hepatocellular carcinoma (HCC). Low adiponectin levels have been observed in either NAFLD or nonalcoholic steatohepatitis (NASH) conditions. However, the effects on leptin as well as the mechanisms of action of adiponectin are unknown. In cirrhosis, circulating adiponectin is elevated. Adiponectin through protein tyrosine phosphatase 1B (PTP1B) prevents the leptin-mediated activation of the janus kinase 2 (Jak2)/signal transducer and activator of transcription-3 (STAT3) pathway through suppressors of cytokine signaling 3 (SOCS3) and the activity of focal adhesion kinase (FAK) and focal adhesion (FA). By inducing SOCS3, adiponectin suppresses leptin activity. Adiponectin can provoke hepatic stellate cells (HSC) apoptosis and leads to the loss of α-smooth muscle actin (α-SMA) proteins in HSCs and suppresses HSC proliferation and α collagen biosynthesis. Adiponectin-mediated prevention of leptin signaling downregulates tissue inhibitor of metalloproteinase 1 (TIMP-1) activity and increases matrix metalloproteinase 1 (MMP-1) to degrade fibrillar collagen in matrix. Adiponectin increases apoptosis of HCC cells via activation of caspase-3, and c-Jun N-terminal kinase (JNK). Adiponectin inhibits leptin-induced proliferation of HCC via blockade of STAT-3, protein kinase B (AKT) and mammalian target of rapamycin (m-TOR) and shows hepatoprotective functions by blocking angiogenesis and sulfatase 2.

In a choline-deficient mouse NASH model, low levels of adiponectin have been reported [49], although their relevance remains unclear. Adiponectin increased apoptosis of hepatocellular carcinoma (HCC) cells via activation of caspase-3, and increased phosphorylation of c-Jun N-terminal kinase (JNK) [50]. Data obtained from cell cultures and animal models suggest that adiponectin inhibits leptin-induced proliferation of HCC via blocking downstream pathways including STAT-3, AKT and mammalian target of rapamycin (m-TOR) [51]. Adiponectin also provokes the suppression of liver tumor growth and metastasis in mice by inhibiting angiogenesis, and it shows chemoprotective and hepatoprotective functions by blocking sulfatase 2 [52,53]. Although basic mechanistic studies indicate that leptin acts to promote HCC proliferation, migration and invasion [54], clinical observation showed that higher leptin expression was associated with increased HCC survival [55]. Similarly, although experimental studies indicated that adiponectin increased HCC apoptosis and prevented liver tumor growth and metastasis, increased adiponectin has also been associated with both favorable prognosis [56] and reduced HCC survival [57]. Further studies are required to resolve this issue. Nevertheless, several concerns should be considered. Experimental models are needed that simulate the progression of NAFLD to HCC in conditions similar to those in clinical practice, and dependence of the roles of adiponectin and leptin on sex must be considered. Indeed, data analysis by gender shows considerably higher levels of adiponectin and leptin in control group females than in males [58]. Lower levels of adiponectin in males may account for their higher prevalence of liver cancer. Testosterone activates JNK in human and mouse adipocytes, and genetic deletion of JNK1 in mouse adipose tissue leads to higher levels of adiponectin and protection against HCC. Increased AMPK and mitogen-activated protein kinase (p38α) activation levels were detected in females, associated with the higher levels of adiponectin in female mice [59] (Figure 3).

## 5. Pharmacological Strategies Regulating the Action of Adiponectin on Hepatic Damage and Regenerative Failure Associated with Hepatic I/R

In an experimental model of LT, the administration of adiponectin reduced hepatic injury and increased regeneration in both steatotic and non-steatotic grafts submitted to 6 h of cold ischemia [16]. Adiponectin increased resistin, which in turn resulted in PI3K/Akt over-expression [16]. In an experimental model of partial hepatectomy using adiponectin –/– mice, the authors reported that adiponectin accelerated cell cycle progression relative to wild-type mice. Such benefits were mediated by STAT3 signaling and progression through the cell cycle [31].

Pharmacological strategies aimed at modulating the actions of adiponectin are ineffective in non-steatotic livers from Zucker rats undergoing 60 min of warm ischemia, as well as in surgical procedures requiring liver regeneration such as partial hepatectomy with I/R [9]. However, treatment with adiponectin protected non-steatotic livers of Wistar rats subjected to 60 min warm I/R by reducing the inflammatory response [C-C motif chemokine ligand 2 (CCL-2), C-X-C motif chemokine ligand 10 (CXCL-10), intercellular cell adhesion molecule (ICAM)-1 and cytokines such as interleukin (IL)-1β, IL-6 and tumor necrosis factor α (TNF-α)] and hepatocyte apoptosis. Such effects of adiponectin are mediated via AMPK/endothelial nitric oxide synthase (eNOS) [60]. These differences in the effects of adiponectin in studies of warm I/R [9,60] might be explained, at least partially, by the use of different species or different treatments: siRNA of adiponectin versus recombinant adiponectin. The studies using adiponectin siRNA and recombinant adiponectin [9,60] assessed the role of endogenous and exogenous adiponectin, respectively. Thus, similar to what occurs for nitric oxide (NO), the roles of endogenous and exogenous adiponectin might be different. The AMPK signaling pathway is also involved in the actions of adiponectin in an experimental model of chronic intermittent liver hypoxia. Thus, adiponectin supplementation induced AMPK activation which reduced the reactive oxygen species (ROS) generation from endoplasmic reticulum stress, and the activation of the three apoptotic pathways [61]. The benefits of adiponectin against inflammation and apoptosis have also been reported in non-steatotic LT without brain death [62]. The mechanism involved in this protection includes anti-inflammatory effects (evidenced by decreased production of neutrophils and inflammatory cytokines, e.g., TNF-α and nuclear factor kappa-light-chain-enhancer of activated B cells (NF-κB) activation) and anti-apoptosis effects, due to decreased expression of the Fas-associated death domain (Fas) and caspase 3 [62].

Regarding the reported effects of adiponectin on liver regeneration, treatment with the anti-diabetic drug rosiglitazone, which is thought to act by elevating serum adiponectin, inhibits liver mass recovery [63]. Nonetheless, in our view, the effects of rosiglitazone on liver regeneration might be explained by mechanisms other than adiponectin upregulation. The results based on adiponectin –/– mice in an experimental model of partial hepatectomy indicate the benefits of adiponectin for liver regeneration [31]. However, these results based on knockout animals might be different from those observed with adiponectin treatment, due to the different effects of endogenous and exogenous adiponectin. It is still unknown whether strategies aimed at increasing adiponectin levels might be useful in NAFLD patients undergoing hepatectomy to improve regenerative failure. Indeed, the over-expression of hepatic adiponectin in patients with NAFLD undergoing hepatectomy is elicited by excessive stimulation of the proinflammatory cytokine, TNF-α, which exerts negative effects on liver regeneration. The relevance of such high adiponectin levels remains to be clarified [17]. Moreover, it has been reported that hepatic accumulation of systemically derived fat from adipose tissue in non-steatotic livers undergoing partial hepatectomy promotes liver regeneration [64]. Interestingly, recent data suggest an association between intrahepatic fat content and adiponectin levels in patients undergoing liver resection [65]. In preclinical studies based on NAFLD undergoing partial hepatectomy, the expression of PPARα is tightly regulated by AdipoR2 axis, whereas the expression of AMPK is a downstream molecule of AdipoR1 axis [17]. Thus, the action of adiponectin depends on the type of receptor as well as on the type of liver and surgical conditions (Table 1).

## 6. Pharmacological Strategies to Regulate Adiponectin Action in Liver Diseases and the Absence of Hepatic I/R

In a study using rats fed ethanol, the anti-inflammatory effects of adiponectin were mediated by regulation of toll-like receptor 4 (TLR4) signaling via the myeloid differentiation primary response gene 88 (MyD88) and TIR-domain-containing adapter-inducing interferon-β (TRIF) pathways [66] (Table 2). In AdipoR1- and AdipoR2-knockout obese mice, treatment with adiponectin decreased the levels of mitochondrial lipid peroxidation since it upregulated uncoupling protein 2 (UCP2), catalase and superoxide dismutase 1 (SOD1) in the liver [67].

It has also been suggested that one explanation for the absence of a NAFLD phenotype might be upregulation of adiponectin, which activates the AMPK–forkhead box protein O (FOXO) signaling axis and probably overrides detrimental oxidative stress and JNK signaling [68] (Figure 1).

Decreased serum levels of adiponectin and decreased gene expression of ileum fibroblast growth factor 15 (FGF15) have been reported in chronic ethanol fed mice, whereas elevated levels of circulating adiponectin and FGF15 protected against inflammation and liver damage [69,70]. These results suggest that the adiponectin–FGF15/19 axis participates in the regulation of ethanol-induced inflammation in mouse liver [69,70]. Serum levels of adiponectin are inversely associated with hepatic bile acid synthesis, serum bile acid levels and hepatocellular injury in NAFLD patients [71]. Additionally, adiponectin directly regulates genes related to bile acid homeostasis such as cholesterol 7 alpha-hydroxylase (Cyp7a1) [71]. Given that both FGF15/19 and adiponectin can regulate bile acid homeostasis, this could partially explain the protective effect of adiponectin–FGF15/19 signaling against ethanol- induced liver injury [72] (Figure 1). These observations might be of relevance for liver surgery given the dysregulation of the farsenoid-X receptor (FXR)-FGF15 pathway in the gut–liver axis in different liver diseases. Whether adiponectin exerts its action directly on the liver or on the intestine (the source of FGF15 generated by ileal FXR) remains to be elucidated. This is scientifically and clinically relevant to the search for the best route of adiponectin administration. It is well known that, under brain death conditions, the gut and hepatic blood flow is reduced and this makes drug delivery to the appropriate site of action and at the optimal concentration difficult. In addition, a recent study by our group of steatotic and non-steatotic LT from brain-dead donors indicates the critical role of the gut–liver axis and the relevance of FGF15/19 in the pathogenesis of hepatic I/R injury and regenerative failure [73].

Pharmacological intervention aimed at elevating adiponectin levels have been reported as promising for the treatment and progression of NAFLD [74]. Thus, one study in patients with NASH shows that increases of adiponectin produced by pioglitazone are related to improvements in steatosis, inflammation and fibrosis, thereby confirming the crucial role of adiponectin in this pathology [75]. Clinical and experimental studies of NASH show that PPAR agonists or vitamin E are potential therapies in NAFLD/NASH since they upregulate adiponectin levels [76,77]. Treatment with PPARγ, such as rosiglitazone, increases the levels and sensitivity of adiponectin receptors [78]. Hume et al. indicated that after 16 weeks of supplementing overweight children with prebiotic fiber, there was a significant increase in adiponectin, while leptin levels did not change [79]. Supplementation with the probiotic *Lactobacillus gasseri* (SBT 2055) reduced abdominal visceral fat and increased serum adiponectin in obese people [80]. Thus, to date, pharmacological evidence suggests that metabolic improvements induced by anti-obesity drugs (orlistat, sibutramine and rimonabant), insulin sensitizers (metformin and thiazolidinediones) and endocannabinoid receptor antagonists could be attributed, at least in part, to the induction of high plasma levels of adiponectin [81,82,83,84]. The results show an increase in plasma adiponectin, ghrelin and leptin levels, as well as insulin sensitivity, four weeks after melatonin administration in a cohort of patients with NASH [82]. Those authors hypothesized that melatonin could improve the key pathogenetic factors associated with NAFLD, namely insulin resistance and hypoadiponectinemia, and in turn confer protection against inflammation and oxidative stress [82,85,86] (Table 2).

In our view, the interpretation of these results of the treatments that increase adiponectin levels should be taken into account because of the lack of specificity in the modulation of adiponectin action. The difficulties producing functionally active recombinant adiponectin are well known, as the molecule is subjected to extensive post-translational modifications and is secreted in complex multimers [87,88]. For this reason, in our opinion, adiponectin analogs, such as osmotin (a ligand for the yeast homolog of the adiponectin receptor) [89], might provide a therapeutic alternative. Another way is indirect upregulation of innate adiponectin expression and secretion through administration of appropriated therapeutic agents [87,88].

Despite the benefits of drugs regulating adiponectin action mentioned above, some of them have shown potential side effects; for instance, weight gain and fat redistribution from the central area to the lower body and also hepatotoxicity are reported following pioglitazone treatment [90,91,92,93,94]. One study suggested that PPARγ activity in liver of mice leads to storage of lipid in the liver [95,96]. Different data in rodent models showed that rosiglitazone exacerbates hepatic steatosis [95,96,97]. Thiazolidinediones causes increased risk of bladder cancer, weight gain, edema, cardiovascular complications and bone loss [95]. There are several concerns about using vitamin E, such as increase in the relative risk of hemorrhagic stroke and prostate cancer [98,99,100]. One study on ob/ob mice indicated that rosiglitazone dramatically increases liver steatosis [78,97]. Furthermore, it has been reported that weight and fat gain, water retention, cardiovascular toxicity, early signs of hypertrophic cardiomyopathy, hepatosteatosis and hepatotoxicity are side effects of rosiglitazone treatment [101,102]. In addition to mild unpleasant gastrointestinal side effects that are commonly reported with orlistat use, increased risk of serious hepatic events is another concern of using this medication [103]. Common side effects of sibutramine include nausea, headache and increased risk of myocardial infarction and stroke [104]. The results of one meta-analysis show that treatment with rimonabant is associated with psychiatric and neurologic adverse events [105]. Therefore, more studies are needed before future application of these drugs in the clinical practice.

## 7. Adiponectin as a Prognostic Factor in Liver Diseases

In rodents with obesity induced by a high fat diet and in ob/ob mice, adiponectin levels in plasma were decreased [106,107,108]. In patients with NAFLD, low levels of adiponectin are closely associated with the degree of hepatic steatosis, necroinflammation and fibrosis [109,110]. Adiponectin plays an important role in the progression of simple liver steatosis to NASH [111,112,113,114]. Thus, different studies suggest the use of serum adiponectin levels as a diagnostic measure of the necro-inflammatory grade and fibrosis in NAFLD, as well as it being a potential NAFLD therapeutic target [115]. However, multivariate regression analysis identifies decreased adiponectin as an independent predictor of liver steatosis in obese individuals [116]. This is in line with several large prospective studies of NAFLD patients, which investigated the role of serum adiponectin as a prognostic factor in NAFLD [117,118]. Thus, in a meta-analysis published in 2018 of 122 studies, the importance of adiponectin levels in the diagnosis of NAFLD was limited [119]. Consequently, it has been reported that more sensitive and specific diagnostic methods are needed to diagnose NAFLD [120]. In our view, and in line with reports by other authors [103], the combination of different cytokines might be the best prognostic factor in liver diseases. Indeed, lower serum adiponectin and resistin concentrations and higher serum RBP4 concentrations were evident in children with advanced liver steatosis. In addition, the same authors indicated that adiponectin, resistin and RBP4 levels could be useful for differentiating patients with advanced liver steatosis from those with mild steatosis [121]. There is a reverse association between serum adiponectin and the presence of NAFLD, which is positively associated with visfatin, IL-6 and TNF-α. It has been reported that an increased probability of NASH would be followed by decreased levels of serum adiponectin and elevated levels of circulating visfatin, IL-8 and TNF-α [115]. This is in line with studies indicating that the evaluation of adiponectin level by itself is insufficient for a diagnosis of NAFLD/NASH and their progression [115,121]. Shimada et al. reported that 90% of patients with early-stage NASH could be predicted by a combined evaluation of serum levels of adiponectin, homeostatic model assessment for insulin resistance (HOMA-IR) score and serum type IV collagen 7S level [122]. Likewise, in patients with cirrhosis, higher levels of adiponectin are associated with liver dysfunction and worse prognosis [123,124,125,126]. Moreover, it has been reported that adiponectin is an independent predictor of overall survival in HCC patients [127,128].

All these observations indicate the importance of future research to elucidate whether adiponectin or a combination of adipocytokines in serum is a useful diagnostic marker in NAFLD/NASH as well as in hepatic resections and LT. Indeed, to the best of our knowledge, no studies have evaluated in detail the potential association between serum adiponectin levels and the hepatic steatosis, damage and regenerative failure associated with liver surgery (Table 3).

Note: ↑, increase; ↓, decrease; Acrp30, Adiponectin; AdipoR2, adiponectin receptor type 2; ALD, alcoholic liver disease; ALT, alanine aminotransferase; HCC, hepatocellular carcinoma; HMW, high molecular weight; IL, interleukin; LT, liver transplantation; MMW, middle molecular weight; NAFLD, nonalcoholic fatty liver disease; NASH, nonalcoholic steatohepatitis; TNF-α, tumor necrosis factor alpha.

## 8. Conclusions and Perspectives

The benefits of adiponectin in terms of obesity, inflammation and regeneration may not apply in all I/R-dependent hepatic surgical scenarios. The role of adiponectin and the signaling pathways involved in its action depend on the surgical conditions, donor and type of liver subjected to surgery. All of this reveals the difficulties in establishing potential targets for the application of adiponectin in clinical practice: if the same pharmacological strategies are applied indiscriminately to steatotic and non-steatotic livers, the effects may be very different.

Specific drugs that regulate the action of adiponectin, research into the structure of adiponectin receptors, identification of molecules downstream of AdipoR1/2 and strategies to enhance adiponectin receptor activity constitute promising approaches to the treatment of NAFLD/NASH [129]. The potential applications of drugs that specifically regulate adiponectin are numerous in liver surgery, which in turn can lead to increasing the number of organs suitable for LT, and may provide a novel therapeutic approach to hepatic resection of tumors. Meanwhile, it is difficult to produce functionally active recombinant adiponectin because the molecule is subjected to extensive posttranslational modifications and secreted in complex multimers. In addition, each type of adiponectin isoform performs different actions, activating specific signaling pathways, and the role of adiponectin also depends on its receptors. Moreover, the types of adiponectin isoforms and receptors involved in each liver disease should be assessed in detail since the studies reported in the literature are very limited. All the related findings need to be considered if our aim is the application of drugs that regulate the actions of adiponectin in clinical practice related to liver diseases.

Further studies are necessary to elucidate the role of adipose tissue in the changes of adiponectin levels in different liver diseases. Currently, the results reported in the literature are contradictory and there is a lack of appropriate experimental designs including for instance the evaluation of adiponectin in adipose tissue, circulation and liver after lipectomy. The signaling pathways involved in the anti-steatotic effects of adiponectin have been evaluated in liver diseases in the absence of surgery. Whether these signaling pathways are similar in steatotic liver surgery remains to be elucidated. This is clinically important because drugs that regulate anabolic and catabolic effects (as in the case of adiponectin) require a certain pretreatment time before they exert their effects, and this is a major problem in LT from brain-dead or cardiac-arrest donors. The potential role of adiponectin in connecting the intestine and liver is scientifically and clinically interesting, and so is its role in regulating FGF15/19 signaling, which is of potential interest due to the benefits on bile acid homeostasis, a critical problem in liver diseases including NASH, cirrhosis and LT. However, appropriate experimental models are required to elucidate the relevance of adiponectin-FGF15/19 in liver diseases and surgery. This is also the case with the effects of adiponectin in fibrosis, since the results are mainly based on in vitro studies, far removed from clinical practice.

Different clinical results have been reported in relation with the levels of adiponectin and the pathological characteristics of NAFLD/NASH and its progression. In the context of hepatic resections and LT, the research into prognostic factors are of clinical and scientific relevance for the treatment of patients but the reported results are very limited and inconclusive. In our view, whether the levels of adiponectin reported in liver diseases might be the result of or the reason for the pathology, itself remains to be elucidated. Potential differences in the levels of adiponectin in liver diseases depending on gender should be considered (they have not been evaluated in detail to date). In addition, although adiponectin might be elevated in liver diseases, it might not exert protection if its receptor is downregulated or the adiponectin signaling pathway is dysfunctional. In addition, in line with other authors, in our view, adiponectin expression may initially be elevated to compensate disease progression, but then higher adiponectin levels could turn out to be ineffective because of an overall deterioration of the patient’s condition [130]. All of these observations should be taken into account for future studies aimed at evaluating whether adiponectin can be considered a useful prognostic factor in different liver diseases in the presence or absence of surgery.

## Figures and Tables

**Table 1:** Effect of strategies that regulate adiponectin action in liver surgery in studies from 2010 to 2020.

Treatment	Isoform and Receptor	Type of Liver and Specie	Surgical Condition	Effect and Signaling Pathways
Adiponectin recombinant (Rat Acrp30) [16]	Not reported	Steatotic and non-steatotic livers from Zucker rats	LT Ischemia: 6 h Reperfusion: 4 h	↓ Hepatic injury and mortality ↑ Ki-67 *Adiponectin-↑ Resistin-↑ PI3K/Akt*
Dietary model [17]	Not reported; AdipoR1 and AdipoR2	Steatotic livers from Sprague-Dawley MCD or HFD rat	Partial (70%) hepatectomy	↓ AdipoR1 and AdipoR2 ↑ Hepatic adiponectin, TNF-α, AMPK AdipoR1-↑ AMPK in MCD and HFD *AdipoR2-↓ PPARα in MCD*
Adiponectin (-/-) [31]	Not reported	Livers from B6.129-Adipoq^tm1Cha^ knockout mice	Partial hepatectomy	↓ Regeneration by controlling cell cycle progression, cytokine signaling and growth factor bioavailability *Adiponectin-↑ STAT3*
Adiponectin recombinant [60]	Not reported	Livers from Wistar rats	Partial warm ischemia Ischemia: 60 min Reperfusion: 6, 24 h	↓ Hepatic injury, inflammatory cell infiltration, IL-1β, IL-6, TNF-α, CCL2, CXCL10, ICAM1, apoptosis *Adiponectin-↑* *AMPK/eNOS*
Adiponectin recombinant and supplementation (Rat gAcrp30) [61]	Not reported	Livers from Wistar rats	Chronic intermittent hypoxia events for 8 h per day for 4 months	↓ Hepatic injury, ROS production, fasting blood glucose, triglycerides *Adiponectin-↑ AMPK*
Adiponectin recombinant (Rat gAcrp30) [62]	Not reported	Livers from Sprague-Dawley rats	LT Ischemia: 30 min Reperfusion: 3, 6, 12, 24 h	↓ Bile duct injury and apoptosis, Fas, caspase 3, TNF-α, NF-κB activation, MPO, IL-6 *Adiponectin-↓* *NF-κB*

↑, increase; ↓, decrease; Acrp30, Adiponectin; AdipoR1/R2, adiponectin receptor type 1/2; And^-/-^, adiponectin knockout; Akt, protein kinase B; AMPK, adenosine monophosphate-activated protein kinase; CCL2, C-C motif chemokine ligand 2; CXCL10, C-X-C motif chemokine ligand 10; eNOS, endothelial nitric oxide synthase; Fas, Fas-associated death domain; gAcrp30, globular adiponectin; h, hours; HFD, high fat diet; ICAM1, intercellular cell adhesion molecule 1; IL, interleukin; Ki-67, antigen Ki-67, a marker of proliferation; LT, liver transplantation; MCD, methionine-choline deficiency; min, minutes; MPO, myeloperoxidase; NF-κB, nuclear factor kappa-light-chain-enhancer of activated B cells; PPARα, peroxisome proliferator-activated receptor alpha; PI3K, phosphoinositide 3-kinase; ROS, reactive oxygen species; STAT3, signal transduce and activator of transcription 3; TNF-α, tumor necrosis factor alpha. The signaling pathway is described in italics.

**Table 2:** Effect of strategies that regulate adiponectin in liver diseases in the absence of surgery in studies from 2010 to 2020.

Treatment	Isoform and Receptor	Type of Liver and Specie	Surgical Condition	Effect and Signaling Pathways
Adiponectin recombinant (Human gAcrp30) [66]	HMW and Adipo R1 and AdipoR2	KC and RAW 264.7 macrophages from Wistar rats with chronic ethanol-feeding	Cell culture	↓ TLR4 (/MyD88), IFN-β, CXCL10 *Adiponectin–↓ TLR4*
CHIP (-/-) [68]	Not reported; AdipoR1 and AdipoR2	Livers from CHIP knockout mice	Cell culture	↓ Oxidative stress and JNK ↑ Adiponectin, AdipoR1, AdipoR2, AMPK and FOXO *Adiponectin-↑* *AMPK-FOXO*
mLipin-1 (-/-) (Human gAcrp30) [69]	HMW; AdipoR1 and AdipoR2	Livers from mLipin-1 knockout mice with chronic ethanol-feeding	NA	↓ Hepatic injury, inflammation, NF-κB, ↑ Adiponectin, AdipoR1, AdipoR2 and FGF15 *Adiponectin-↑* *FGF15 signaling*
mNT (-/-) [70]	Not reported	mNT knockout mice with chronic ethanol-feeding	NA	↓ Hepatic injury, NF-κB, oxidative stress ↑ Adiponectin, FGF15, Sirt1 *Adiponectin-↑* *FGF15 signaling*
Pioglitazone [75]	Not reported	NASH patients	NA	↓ Hepatic steatosis and necroinflammation ↑ Adiponectin *Adiponectin-↓* *NF-κB/JNK*
Rosiglitazone [78]	HMW; AdipoR1 and AdipoR2	Livers from C57BL/6J mice with ethanol-feeding	NA	↓ Hepatic injury, steatosis, lipogenesis ↑ Adiponectin and hepatic AdipoR1/R2 *Adiponectin–↑* *SIRT1-AMPK*
Prebiotic fiber supplementation [79]	Not reported	Children patients with overweight and obese	NA	↑ Adiponectin and ghrelin *Not reported adiponectin signaling pathway*
Probiotic *Lactobacillus gasseri* (SBT2055) [80]	HMW, MMW, LMW and not reported receptor	Obese patients	NA	↓ Abdominal visceral fat ↑ HMW in obese and control group ↑ MMW only in control group *Not reported adiponectin signaling pathway*
Melatonin [82]	Not reported	NASH patients	NA	↓ HOMA-IR ↑ Adiponectin, leptin and ghrelin *Not reported adiponectin signaling pathway*
Orlistat [84]	Not reported	NAFLD patients	NA	↓ Fatty infiltration, periostin, TNF-α ↑ Adiponectin *Not reported adiponectin signaling pathway*

↑, increase; ↓, decrease; AdipoR1/R2, adiponectin receptor type 1/2; AMPK, adenosine monophosphate-activated protein kinase; CXCL10, C-X-C motif chemokine ligand 10; CHIP, C-terminus of Hsc70-interacting protein; FGF15, fibroblast growth factor 15; gAcrp30, globular adiponectin; FOXO, forkhead box protein O; HMW, high molecular weight; HOMA-IR, homeostatic model assessment for insulin resistance; IFN-β, interferon beta; JNK, c-Jun N-terminal kinase; KC, Kupffer cells; LMW, low molecular weight; mLipin-1, myeloid cell-specific lipin-1; MMW, middle molecular weight; mNT, mitoNEET; MyD88, myeloid differentiation primary response gene 88; NA, not apply; NAFLD, nonalcoholic fatty liver disease; NASH, nonalcoholic steatohepatitis; NF-κB, nuclear factor kappa-light-chain-enhancer of activated B cells; Sirt1, sirtuin-1; TLR4, toll-like receptor 4; TNF-α, tumor necrosis factor alpha. The signaling pathway is described in italics.

**Table 3:** Association between adiponectin levels and pathological characteristics in different liver disease.

Disease	Subjects (Etiology)	Adiponectin Levels	Effect
Alcoholic liver disease (ALD) [58]	147 patients	18.69 ALD 6.38 control	↑ Adiponectin (Acrp30) associated with advanced liver dysfunction and ALD complications
NAFLD [112]	63 patients	4.26 ± 2.71 µg/mL NAFLD 5.85 ± 3.74 µg/mL controls	↓ Three isoforms of adiponectin. HMW and MMW adiponectin involved in the pathogenesis and progression of NAFLD
NAFLD [113]	315 patients (129 mild, 145 moderate, 41 severe)	13.6 ± 3.3 µg/mL mild 12.4 ± 3.7 µg/mL moderate 11.6 ± 3.5 µg/mL severe	↑ adiponectin correlated with a decreased risk of developing type 2 diabetes
NAFLD [114]	232 patients (45 cirrhosis, 71 viral hepatitis, 64 NAFLD, 52 others)	18.6 ± 14.5 µg/mL cirrhosis 8.4 ± 6.1 µg/mL without cirrhosis 4.8 ± 3.5 µg/mL NAFLD 9.1 µg/mL controls	Adiponectin correlate positively with markers of hepatic fibrosis. ↓ Adiponectin in NAFLD and ↑ in cirrhosis
NAFLD [115]	70 patients	8.14 ± 2.91 mg/L NAFLD 13.63 ± 2.88 mg/L controls	↓ Adiponectin and ↑ visfatin, IL-6, TNF-α associated with increased NAFLD
NAFLD [117]	52 patients	3.9 (2.5 – 6.2) µg/mL	Adiponectin, TNF-α, IL-6, leptin were not associated with disease progression
NAFLD [118]	147 patients	9.6 ± 4.1 µg/mL NAFLD 14.0 ± 10.1 µg/mL without NAFLD	↓ Adiponectin associated with NAFLD
NAFLD [120]	71 children patients (37 with NAFLD, 14 with NASH, 20 without NAFLD)	13.15 ± 5.33 ng/mL NAFLD 12.64 ± 5.54 ng/mL NASH	Adiponectin levels were similar in patients with and without NAFLD. ↑ AdipoR2 in NAFLD and is a noninvasive marker for diagnosis
NAFLD [121]	148 children patients (63 steatosis, 12 steatosis and ↑ ALT, 85 without steatosis)	2.7 ± 0.7 µg/mL steatosis 2.5 ± 0.4 µg/mL NAFLD 4.7 ± 1.1 µg/mL without steatosis	↓ Adiponectin were negatively correlated with ALT activity
Cirrhosis [123]	122 patients	21.59 µg/mL cirrhosis 12.52 µg/mL controls	↑ Adiponectin associated with ↑ liver dysfunction and worse prognosis
Cirrhosis [124]	70 patients (40 cirrhosis, 30 cirrhosis and cholestasis)	15.1 ± 12.1 µg/mL cirrhosis 21.28 ± 10.2 µg/mL cirrhosis with cholestasis 4.7 ± 4.48 µg/mL controls	↑ Adiponectin shows correlation with degree of hepatocellular injury and cholestasis; but not with parameters of body composition or metabolism
Cirrhosis [125]	140 patients	13.050 ng/mL	Adiponectin was an independent predictor of overall survival in HCC patients
Cirrhosis [126]	40 patients with non-diabetic alcoholic cirrhosis	10.23 µg/mL	↑ Adiponectin associated with shorter survival in the univariate analysis but not in the multivariate analysis
Cirrhosis [127]	248 patients with compensated viral hepatitis C cirrhosis	16.5 ± 15.3 µg/mL cirrhosis	Adiponectin was not related to HCC, liver-related death or LT during follow-up
Cirrhosis [128]	90 patients with hepatitis C-related liver cirrhosis (61 with, 29 without)	5.213 ± 3.840 µg/mL cirrhosis with HCC 9.000 ± 2.234 µg/mL cirrhosis without HCC	↓ Adiponectin levels associated with HCC; a biomarker of HCC

Note: ↑, increase; ↓, decrease; Acrp30, Adiponectin; AdipoR2, adiponectin receptor type 2; ALD, alcoholic liver disease; ALT, alanine aminotransferase; HCC, hepatocellular carcinoma; HMW, high molecular weight; IL, interleukin; LT, liver transplantation; MMW, middle molecular weight; NAFLD, nonalcoholic fatty liver disease; NASH, nonalcoholic steatohepatitis; TNF-α, tumor necrosis factor alpha.

## Abbreviations

ACC	Acetyl-CoA carboxylase
AdipoQ	Adiponectin, C1Q and collagen domain containing
AdipoR1/R2	Adiponectin receptor type 1/2
And^-/-^	Adiponectin knockout
AICAR	Aminoimidazole-4-carboxamide ribonucleoside
Akt	Protein kinase B
ALD	Alcoholic liver disease
ALT	Alanine aminotransferase
AMPK	Adenosine monophosphate-activated protein kinase
Ang	Angiotensin
APPL1	Adaptor protein
ATF6	Activating transcription factor 6
ATP	Adenosine triphosphate
CCL2	C-C motif chemokine ligand 2
CHIP	C-terminus of Hsc70-interacting protein
CPT-1	Carnitine palmitoyl- transferase 1
CRP	C-reactive protein
CXCL-10	C-X-C motif chemokine ligand 10
Cyp7a1	Cholesterol 7 alpha-hydroxylase
EC	Endothelial cell
eNOS	Endothelial nitric oxide synthase
ER	Endoplasmic reticulum
ET	Endothelin
FA	Focal adhesion
FAK	Focal adhesion kinase
Fas	Fas-associated death domain
Fe	Iron
FGF2	Fibroblast growth factor 2
FGF15/19	Fibroblast growth factor 15/19
FOXO	Forkhead box protein O
FXR	Farsenoid-X receptor
gAcrp30	Globular adiponectin
GSH	Glutathione
h	Hours
HCC	Hepatocellular carcinoma
HFD	High fat diet
HMW	High molecular weight
HO-1	Heme oxygenase-1
HOMA-IR	Homeostatic model assessment for insulin resistance
HSC	Hepatic stellate cells
I/R	Ischemia/reperfusion
ICAM	Intercellular cell adhesion molecule
IFN	Interferon
IL	Interleukin
IRE1	Inositol-requiring enzyme 1
Jak2	Janus kinase 2
JNK	c-Jun N-terminal kinase
KC	Kupffer cells
Ki-67	Antigen Ki-67, a marker of proliferation
KO	Knockout
LCN2/SAA1	Lipocalin-2/serum amyloid A1
LKB1	Liver kinase B1
LMW	Low molecular weight
LT	Liver transplantation
MCD	Methionine-choline deficiency
min	Minutes
mLipin-1	Myeloid cell-specific lipin-1
MMP-1	Matrix metalloproteinase 1
MMW	Middle molecular weight
mNT	MitoNEET
MPO	Myeloperoxidase
m-TOR	Mammalian target of rapamycin
MyD88	Myeloid differentiation primary response gene 88
NAFLD	Nonalcoholic fatty liver disease
NASH	Nonalcoholic steatohepatitis
NF-κB	Nuclear factor kappa-light-chain-enhancer of activated B cells
NO	Nitric oxide
O_2_	Superoxide
ONOO-	Peroxynitrite
p38	Mitogen-activated protein kinase p38
PDGF-BB	Platelet-derived growth factor-BB
PERK	Protein kinase-like endoplasmic reticulum kinase
PI3K	Phosphoinositide 3-kinase
PPAR	Peroxisome proliferator-activated receptor
PTP1B	Protein tyrosine phosphatase 1B
RBP4	Retinol binding protein 4
ROS	Reactive oxygen species
siRNA	Silent small interfering RNA
SIRT1	Sirtuin-1
α-SMA	α-smooth muscle actin
SOCS3	Suppressors of cytokine signaling 3
SOD	Superoxide dismutase
SREBP-1c	Sterol regulatory element-binding protein 1c
STAT3	Signal transducer and activator of transcription 3
TGF-β	Tumor growth factor beta
TIMP-1	Tissue inhibitor of metalloproteinase 1
TLR4	Toll-like receptor 4
TNF-α	Tumor necrosis factor alpha
TRAF6	TNF receptor-associated factor 6
TRIF	TIR-domain-containing adapter-inducing interferon-β
UPC2	Uncoupling protein 2
UPR	Unfolded protein response
X/XOD	Xanthine/Xanthine oxidase
XOD	Xanthine oxidase
